# Emergency medical service personnel' post-traumatic stress disorder and psychological detachment: The mediating role of presenteeism

**DOI:** 10.3389/fpubh.2023.1030456

**Published:** 2023-03-07

**Authors:** Dandan Liao, Yanqiong Long, Tao Yu, Xiaoyan Kang, Shulai Liu, Jin Yan, AiDi Zhang

**Affiliations:** ^1^Xiangya Nursing School, Central South University, Changsha, Hunan, China; ^2^Department of Emergency Center, The Third Xiangya Hospital, Central South University, Changsha, Hunan, China; ^3^Department of Nursing, Third Xiangya Hospital, Central South University, Changsha, Hunan, China; ^4^Department of Critical Care Medicine, The Third Xiangya Hospital, Central South University, Changsha, Hunan, China

**Keywords:** PTSD, presenteeism, mediation, emergency, China, structural equation modeling

## Abstract

**Background:**

Emergency medical service personnel are subjected to various stressors, which makes them more likely to develop post-traumatic stress disorder symptoms. Studies have shown that psychological detachment and presenteeism play a role at the level of post-traumatic stress disorder. There is no study to examine the relationship between psychological detachment, presenteeism, and post-traumatic stress disorder among emergency medical service personnel.

**Objective:**

The main objective of the study is to investigate the effects of presenteeism in explaining the relationship between psychological detachment and post-traumatic stress disorder among emergency medical service personnel.

**Design:**

A cross-sectional study was conducted among 836 emergency medical service personnel in 51 counties and cities in Hunan Province, China.

**Methods:**

They were anonymously investigated by using the Impact of Event Scale-Revised (IES-R), the Stanford Presenteeism scale-6 (SPS-6), and the Psychological Detachment Scale. Statistic description, univariate analysis, pearson correlation, and structural equation model were adopted to analyze the data.

**Results:**

The mean score of IES-R, SPS-6, and the psychological detachment scale were 22.44 ± 16.70, 15.13 ± 4.20, and 11.30 ± 4.24. Post-traumatic stress disorder was positively relevant with presenteeism (*r* = 0.381, *p*< 0.01), but negatively correlated with psychological detachment (*r* = −0.220, *p* < 0.01). And presenteeism partially mediated the association between psychological detachment and post-traumatic stress disorder.

**Conclusions:**

The results show a high prevalence of post-traumatic stress disorder symptoms in EMS personnel, presenteeism can statistically significantly predict post-traumatic stress disorder symptoms. If hospital management can reduce the presenteeism of emergency medical service personnel, this will help them reduce post-traumatic stress disorder symptoms.

## 1. Introduction

Emergency medical service (EMS) personnel are often at increased risk of exposure to dangerous and traumatic events (1) and various stressors (2). Moderate levels of stress were reported by 74.8% of EMS personnel in Iran (3). These pressures place burden on EMS personnel and thus jeopardize the quality of emergency care and effects the outcome of patient treatment (4). Besides, studies have shown that EMS personnel are more likely to experience mental disorders (5). The prevalence of stress disorders among them is not only higher than in the general population (6), but also statistically significantly higher than in other emergency services such as police and firefighters (7). Exposure to job-related stressors can increase the risk for post-traumatic stress disorder (PTSD) (8). Available evidence has suggested that constant exposure to negative stressors may potentially damage a person's physical and mental health (9) and can make individuals more susceptible to PTSD (10).

PTSD is defined as a mental disorder that occurs after individual experiences a traumatic event (11). It consists of three dimensions, which are intrusion, avoidance and hyperarousal. Data from a survey in Polish suggests that PTSD was identified in 40% of EMS personnel (12). The risk of suicide among those with PTSD symptoms is about 6.74 times higher than else, which poses a risk to the individual and society (13). Approximately 5.3% of EMS personnel in Australia reported having had suicidal thoughts in the past 1 year, and 12.3% had considered suicide (14). PTSD may result in negative performance, reduce job satisfaction (15) and increase the willingness to quit a job (16).

Presenteeism is defined as employees who should have taken time off from work for health reasons, but had to go to work, resulting in loss of production (17). Studies have shown that the primary cause of presenteeism is mental health problems, such as depression and tension (18), depression is likely to cause lack of energy (19). In addition, employees with high psychological stress have more presenteeism than normal people (20). It was reported that 59% of pediatric resident physicians in Canada had come to work sick (21). In Shan's study, nurses have a high level of presenteeism (22). There is much evidence showing that presenteeism could lead to tired, low work productivity and other adverse outcomes and affects people's ability to cope with traumatic events, these can lead to PTSD susceptibility (23). To the best of the researcher's knowledge, the relationship between presenteeism and PTSD among EMS personnel has not been examined. We assume that presenteeism as a psychological variable, will affect EMS personnel' s PTSD.

Positive psychology can have a lasting impact on life (24). However, psychological detachment as a popular topic of positive psychology has been attached limited attention to relieve PTSD symptoms. Psychological detachment is a mental state in which an individual is psychologically separated from work (25), it can relieve stress caused by stressors (26). Sonnentag proposed a stressor-detachment model that explained the role of psychological detachment in the stressor–strain process (27). The model shows that when employees are exposed to high stressors, it is more hard for them to separate from work. On the one hand, it plays a buffering role against psychological stress, it can protect individuals in stressful situations (28). On the other hand, it can promote the well-being and work engagement of workers (29), and had a negative predictive effect on turnover intention (30). Psychological detachment is a protective factor in the stressor-stress response relationship (27). Therefore, psychological detachment adjusts pressure and stress responses. However, whether psychological detachment plays a role in PTSD remains unclear.

This study investigates the mental health status of EMS personnel and explores the correlation between PTSD and these factors. It can provide managers a basis for future effective psychological interventions for them to reduce the psychological harm caused during emergency care and improve their life quality. The main purpose was to examine the direct and indirect effects of variables on PTSD among EMS personnel.

This is the first research that aimed to examine the associations between psychological detachment, presenteeism, and PTSD among EMS personnel. Thus, stemming from theory and the reviewed literature, we hypothesized that: (1) Greater psychological detachment would be predictive of lower PTSD symptoms. (2) Presenteeism would have a direct impact on aggravate PTSD symptoms. (3) Presenteeism would mediate the relationship between psychological detachment and PTSD symptoms.

## 2. Methods

### 2.1. Study population and data collection

#### 2.1.1. Study population

At the annual meeting of Hunan EMS personnel, we conducted an electronic questionnaire for the EMS personnel who came to the meeting. A cross-sectional study was conducted among EMS personnel from 51 counties and cities in Hunan Province, China in July 2021 by using the convenience sampling. The inclusion criteria were as follows: (1) age ≥18 years old; (2) those who are had emergency work time ≥1 year; (3) informed consent and cooperation with this investigation. Exclusion criteria were as follows: (1) those who are on sick leave or maternity leave; (2) They are doing other intervention studies; (3) Medical staff on training or rotation. With 32 observed variables and 6 latent variables in this study, we calculate that a minimum sample of 403 people is required to build the structural equation model.

#### 2.1.2. Data collection

We recruited 836 EMS personnel in the study. Questionnaires with potentially from random responding were excluded, the number of usable questionnaires was reduced to 719, the effective response rate of 86%. Questionnaires were self-administered on a mobile phone and pertained to demographic characteristics, psychological detachment, presenteeism, and PTSD. All scales are Chinese scales that have been well-translated and have good reliability and validity. Before the questionnaire was started, participants' oral informed consent was obtained. The investigators informed the participants of the purpose of this study and the principles of voluntarily and anonymously. And no personal identifiers were obtained to ensure confidentiality. This study was approved by the ethical review board of Xiangya School of Nursing, Central South University. Ethics review number: E2021155.

### 2.2. Measurements

#### 2.2.1. General information

The demographic information of EMS personnel was collected, including age, gender, education, working seniority, career, educational background, marital status, monthly income, work unit, educational level, employment form, and diagnosis of chronic diseases.

#### 2.2.2. Impact of events scale-revised

The Impact of Events Scale-Revised (IES-R) was developed by Weiss and Marmar (31) and converted to the Chinese version by Guo et al. (32). The IES-R is one screening instrument for PTSD. It contains 3 dimensions (intrusion, avoidance, and hyperarousal) and 22 items in total. Using a 5-point Likert scale, each entry was assigned with a score ranging from 0 to 4 points. The total IES-R score>34 suggests statistically significant PTSD symptoms (33). Higher scores indicated higher levels of PTSD. The Cronbach's coefficients of the intrusion, avoidance, and hyperarousal sub-scales were 0.933, 0.936, 0.926, respectively.

#### 2.2.3. Stanford presenteeism scale

Stanford Presenteeism Scale (SPS-6) (34) which was originally compiled by Koopman et al. (35). We used it to assess the impact of health problems on an individual's productivity. The participants responded to the items that they have experienced in the last month. It contains six items, containing two dimensions of finishing work (four items) and avoiding distraction (two items scored in reverse). Using a 5-point Likert scale, each entry was assigned with a score ranging from 1 to 5 points. Higher scores indicated the greater the loss of health-related productivity caused by the presenteeism. In this study, Cronbach's coefficient was 0.636.

#### 2.2.4. Psychological detachment scale

There are four items in the psychological detachment scale, a component of the recovery experience questionnaire compiled by Sonnentag and Fritz (36). The Chinese version of this scale was revised by Lu et al. (30), which has been widely used for measuring psychological detachment. It uses a 5-point Likert scale ranging from 1 to 5 points, with higher scores indicating higher levels of individual psychological detachment. The total score of psychological detachment was divided into high and low detachment levels with a cut-off value of eleven. In this study, Cronbach's coefficient was 0.867.

### 2.3. Statistical analyses

Statistical analysis was conducted by software SPSS 24.0 and AMOS 24.0. First, continuous variables are described in terms of mean and standard deviation. *T*-tests and one-way analysis of variance (ANOVA) were used to examine whether statistically significant differences exist in PTSD among groups. Secondly, Pearson's correlation coefficients were used to explore the correlations between three dimensions of IES-R, SPS-6, and the psychological detachment scale. Thirdly, the structural equation modeling was used to determine the hypothetical mediation model, and the relationship between variables was determined by using AMOS 24.0. The goodness of fit of the model was evaluated using the following indices: degrees-of-freedom (df) ratio (x^2^/df), the root mean square error of approximation (RMSEA), the normed fit index (NFI), the comparative fit index (CFI), the relative fit index (RFI), and the incremental fit index (IFI). Fourth, the bootstrapping analysis has been adopted to examine the mediating effect. It was used to estimate the significance of indirect effects by 5,000 bootstraps and 95% confidence interval, if the confidence interval included zero, then the indirect effect was regarded as statistically insignificant. *P*-values < 0.05 were considered statistically significant.

## 3. Results

### 3.1. Demographics of the participants

Table 1 presents the demographic characteristics as well as the results of the univariate analysis of the sample. A total of 719 EMS personnel were enrolled in this study. The average age of the participants was 33.98 years old (SD = 8.134; range: 20–58). The sample was mainly of nurses (57.6%), female (55.2%). Approximately 57.7% of EMS personnel were with the educational level of bachelor degree and high. The majority (68.6) of EMS personnel were married. Nearly nine in tenth (92.5%) of EMS personnel reported with a monthly income of 3,000 RMB and more. Results of the univariate analysis demonstrated that education level, marital status, nature of employment, and disease history were statistically significantly associated with IES-R (*p* < 0.05).

**Table 1:** Descriptives and correlations among the demographic characteristics and IES-R scores.

**Variable**		***n* (%)**	**mean (SD)**	***t*/*F***	***p***
Gender	Male	322 (44.8)	22.67 ± 18.43	0.332	0.740
	Female	397 (55.2)	22.25 ± 15.17		
Age	≤34	413 (57.4)	21.19 ± 16.30	2.848	0.059
	35–44	209 (29.1)	23.78 ± 17.23		
	≥45	97 (13.5)	24.85 ± 16.93		
Educational level	Technical school	304 (42.3)	19.89 ± 16.37	6.297	0.020
	Undergraduate	403 (56.1)	24.36 ± 16.71		
	Master	12 (1.7)	22.67 ± 17.48		
Marital status	Unmarried	201 (28.0)	19.91 ± 14.81	4.066	0.018
	Married	495 (68.8)	23.22 ± 17.33		
	Divorced	23 (3.2)	27.78 ± 16.16		
Income status	<3,000 yuan	54 (7.5)	19.15 ± 18.62	1.3	0.269
	3,001–6,000 yuan	371 (51.6)	22.01 ± 16.50		
	6,001–9,000 yuan	240 (33.4)	23.76 ± 17.02		
	9,001–12,000 yuan	49 (6.8)	21.96 ± 13.87		
	>12,000 yuan	5 (0.7)	31.00 ± 17.55		
Career	Doctor	182 (25.3)	24.15 ± 17.73	1.727	0.160
	Nurse	414 (57.6)	22.47 ± 15.31		
	Driver	116 (16.1)	19.65 ± 19.55		
	Else	7 (1.0)	22.29 ± 13.62		
Nature of employment	Contracts	384 (53.4)	21.21 ± 15.84	3.399	0.017
	Human agency	39 (5.4)	29.59 ± 23.29		
	Authorized	269 (37.4)	23.29 ± 15.99		
	Else	27 (3.7)	21.19 ± 21.68		
Diseases history	Yes	419 (58.3)	25.65 ± 17.33	6.434	0.000
	No	300 (41.7)	17.95 ± 14.66		

### 3.2. Correlations of study variables

Table 2 presents the basic descriptive data for IES-R, SPS-6, and Psychological Detachment Scale and the results of Pearson's correlation analysis. The mean total scores for IES-R were 22.44 ± 16.70 (range = 0–88), the mean total scores for SPS-6 were 15.13 ± 4.20 (range = 6–30), and the mean total scores for Psychological Detachment Scale were 11.30 ± 4.24 (range = 4–20). The study showed that all of the SPS-6 and Psychological Detachment Scale were statistically significantly associated with IES-R. There was a statistically significant positive correlation between SPS-6 and IES-R (*r* =0.381, *p* < 0.01), which means that the severer the presenteeism, the severer the PTSD; a statistically significant positive correlation between psychological detachment Scale, and IES-R (*r* = −0.220, *p* < 0.01), which means that people who have higher psychological detachment will have fewer PTSD. The above results support the hypothesis of the relationship among the three, and we conducted an intermediary model to further validate the hypothesis.

**Table 2:** Means, standard deviations, and correlations among main variables of study (*N* = 719).

	**X ±S**	**IES-R**	**Intrusion**	**Avoidance**	**Hyperarousal**	**SPS-6**	**Pychological detachment**
IES-R	22.44 ± 16.70	1.00					
Intrusion	6.38 ± 4.76	0.954^**^	1.00				
Avoidance	7.69 ± 6.04	0.967^**^	0.905^**^	1.00			
Hyperarousal	8.37 ± 6.57	0.960^**^	0.869^**^	0.882^**^	1.00		
SPS-6	15.13 ± 4.20	0.381^**^	0.323^**^	0.342^**^	0.418^**^	1.00	
Psychological detachment	11.30 ± 4.24	−0.220^**^	−0.210^**^	−0.166^**^	−0.236^**^	−0.060^*^	1.00

The ^*^ symbol indicates *p* value <0.05.

The ^**^ symbol indicates *p* value <0.01.

### 3.3. Test of the hypothesized model and parameter estimates

The SEM analysis was conducted with AMOS 24.0 software. In Table 3, the modification indices were used to correct the structural model. The modification indices showed that the structural model with good fit was CMIN/df = 1.402, RMSEA = 0.024, NFI = 0.998, CFI = 1.000, RFI = 0.995, IFI = 1.000, *p* = 0.240. fitted with the SEM criteria (χ2/df <5, NFI, CFI, RFI, IFI >0.90, and RMSEA <0.08) (37).

**Table 3:** The comparison of the parameters of the model before and after the modification.

	**CMIN/df**	**RMSEA**	**NFI**	**CFI**	**RFI**	**IFI**	***p***
Before revision	12.697	0.128	0.980	0.982	0.951	0.982	0.000
After revision	1.402	0.024	0.998	1.000	0.995	1.000	0.240

**Figure 1:**
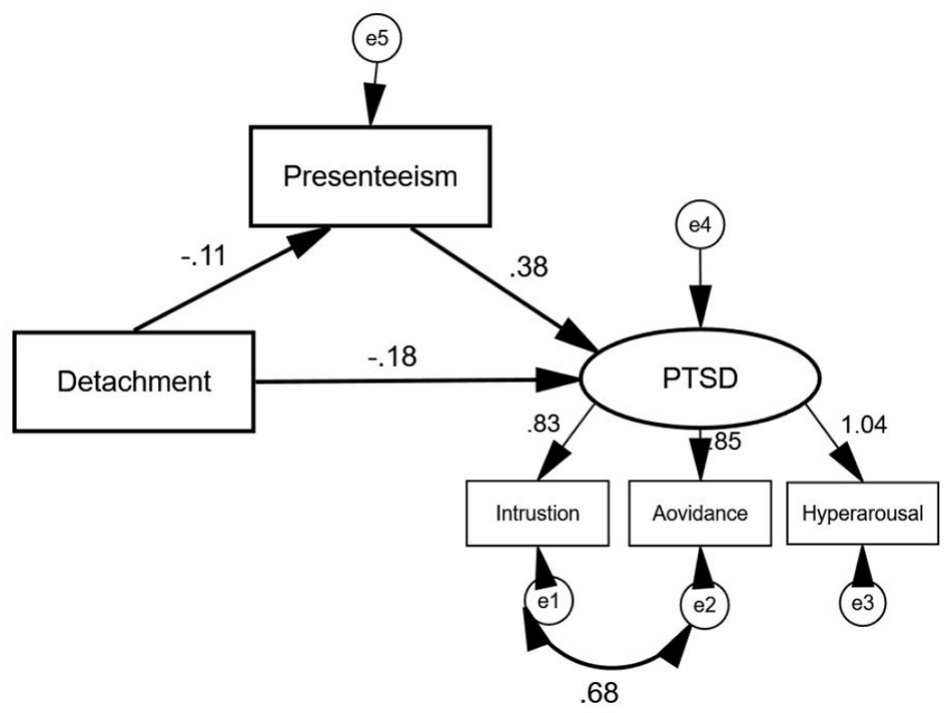
The structural equation model of Psychological detachment, SPS-6, and IES-R.

As is indicated in Figure 1. The mediating effect of SPS-6 between the relationship of psychological detachment and IES-R was confirmed with structural equation model. The dependent variable was the IES-R, with the psychological detachment as the independent variable and SPS-6 as the mediator in structural equation model.

Table 4 presents the bootstrapping analysis with 5,000 iterations has been adopted to examine the mediating effect of presenteeism on psychological detachment and PTSD. The pluggable unit of PROCESS in SPSS, which is developed by Hayes (38), was applied to analyze the mediation effect. In Table 4, zero is not included in the 95% confidence interval, then the direct, indirect, and total effects are statistically significant. Therefore, presenteeism plays a partial mediating role in the relationship between psychological detachment and PTSD, the indirect effects accounted for 18.18% of the total effect.

**Table 4:** Bootstrapping effect and 95% confidence intervals (CI) for the mediating model.

**Path**	**β (standardization)**	**Bootstrap 95% CI**	***p***
Total effect (c)	0.22	−1.15 to −0.58	<0.001
Direct effect (c')	−0.18	−0.98 to −0.45	<0.001
Indirect effect (a × b)	0.04	−0.26 to −0.04	<0.001

## 4. Discussion

This study shows that the IES-R score was 22.24 ± 16.59, and the PTSD symptoms detection rate of 22.25% among EMS personnel. The detection rate was statistically significantly higher than medical personnel in ICU (13%) (39), and pediatrics nurses (20.1%) (40). This may be related to the different study populations, where EMS personnel has heavy workloads, patients' conditions, and work stress that predispose them to PTSD. This is consistent with the results of other studies, Petrie also found that EMS personnel has a prevalence of PTSD considerably higher than general population (6). The dimensions, in descending order of score, are intrusion, hyperarousal, avoidance. The highest intrusion response score indicates that EMS personnel is more frequently disturbed by stressful and traumatic events in the workplace. We can concluded that the score is higher for those with a bachelor's degree than those with less than a bachelor's degree, higher for those with a staffing agency than those with an establishment like employment, higher for those with a history of illness than those without a history of illness and higher for those married than those unmarried. Contrary to Carleton's study, married people are less likely to be screened for mental health disorders than single people (5). In our study, there were no gender differences between males and females. But in Brivio's research, female' scored higher than male on the total score as well as on all dimensions (41). This may be related to the context of her research, which is the psychological changes of the general public in Italy after the outbreak of the new crown epidemic.

Psychological detachment scores were at a moderately low level. Psychological detachment scores were lower than Poulsen's findings for radiation therapists and oncology nurses (42). This indicates that it is difficult for EMS personnel to separate physically and psychologically from their work after hours and that they are still affected by their work. There is room for improvement in the level of the psychological detachment of EMS personnel.

Presenteeism scores were at a high level, but less than Skela's findings for Slovenian nurses (43). No investigation of presenteeism of EMS personnel is available. On the one hand, it indicates that EMS personnel has physical and psychological problems, with 56.9% of the study participants indicating that they have a history of illness; On the other hand, it may be related to the traditional Chinese culture. Some studies say that Chinese employees have statistically significantly higher stress levels and attendance behaviors than British employees, which can be explained by traditional Confucian values and collectivism that emphasizes hard work and endurance (44). This may be related to their heavy workload and shortage of human resources. The above results suggest that EMS personnel are in the poor psychological condition.

This study showed that the psychological detachment of EMS personnel was negatively correlated with PTSD (*r* = −0.220, *p* < 0.01), the higher the level of psychological detachment, the lower the probability of suffering from PTSD. This result is consistent with Sonnentag's model (27), which believes that if workers can be mentally separated from work, the negative impact of job demand on body and psychology can be reduced. Research indicates that victims' subjective reactions to assaults can predictors of the incidence of post traumatic stress symptoms (45). Han's study showed that employees' psychological detachment levels were positively correlated with their life satisfaction (46), negatively correlated with depression (47). Therefore, psychological detachment makes EMS personnel experience fewer traumatic events, reduces PTSD and the negative consequences of their work. This study showed that presenteeism was negatively associated with psychological detachment and positively associated with the dimensions of PTSD. This means that the higher the level of presenteeism, the higher the probability of developing PTSD. The Job Demands-Resources model points out that people in disease states need to consume more resources (48), this means that presenteeism will decrease the workforce productivity. This study also found that presenteeism correlated more with intrusion dimensions of PTSD. Intrusion dimensions is the most characteristic manifestation of post-traumatic stress and the primary symptom in the development of stress disorder (49), when people are exposed to scenarios associated with traumatic events, intense psychological distress and physiological reactions occur. Due to work stress, EMS personnel have a higher incidence of presenteeism than other occupations (50) and are more likely to remember traumatic events that occurred after work.

According to the results of the equation model. We demonstrated that implicit presenteeism mediated the relationship between psychological detachment and PTSD. Based on the Job Demand-Resource model, low levels of psychological detachment lead to chronic overconsumption of resources and eventually to resource imbalance, leading to presenteeism. The lack of human resources and the high work pressure have led to EMS personnel having a relatively low level of psychological detachment. This ultimately leads to an increase the probability of being unable to respond to an emergency event and increases the chances of PTSD. Resolution of stress, which increases the incidence of presenteeism. Similarly, physical or psychological discomfort Increased the incidence of presenteeism. This ultimately leads to an increased probability that EMS personnel will not be able to respond to emergencies and increases the chance of PTSD.

Managers need to focus on the relationship between psychological detachment, presenteeism, and PTSD. Improving psychological detachment of EMS personnel reduces the level of presenteeism, thus reducing their PTSD symptoms. We recommend that managers take appropriate measures in terms of both prevention and treatment. (1) Prevention: we should regularly assess the psychological condition of EMS personnel and establish psychological files, providing relevant consultation services to reduce their post-traumatic stress level, which in turn affects the quality of clinical care and the stability of hospital human resources. Likewise, EMS personnel should also experience the rewards and benefits of helping people and enhancing their professional quality of life (51). More importantly, strengthening exercise can effectively alleviate psychological depression symptoms (52). (2) Treatment: Those that have been shown to be effective are mindfulness, Animal-assisted interventions (AAIs) (53), zimbardo time perspective therapy (ZTPI) (54), acceptance and commitment therapy (ACT) (55), digital health (56), critical incident stress debriefing, eye movement desensitization and reprocessing (EMDR), cognitive processing therapy (CPT) (57), etc.

This study has several limitations. First, we collected the cross-sectional data, it could not infer the causes associations among psychological detachment, presenteeism, and PTSD. Secondly, a convenience sampling method was adopted, the samples only came from one province in China. The data collected in this way are not comprehensive enough and the sample is less representative. The last one, in a descriptive investigation using self-reported structured questionnaires, respondents may have underestimated or overestimated the level, behavioral measures should be incorporated into future research.

## 5. Conclusions

The results of the study showed that the prevalence of PTSD among EMS personnel was high, the psychological detachment had a negative effect on PTSD, and presenteeism had a positive effect on PTSD, while psychological detachment was also an important influencing factor of presenteeism. Based on the Stressor-detachment model, this study investigated the effects and pathways of psychological detachment and presenteeism on PTSD in EMS personnel. Therefore, The mental health status of EMS personnel needs attention. This study provides a research base for managers to provide psychological interventions to EMS personnel in the future. Hospital managers should pay attention to the level of psychological detachment and presenteeism of EMS personnel, and then reduce the prevalence of PTSD among them. EMS personnel also need to increase their attention to psychological detachment in order to cope with emergency work, thereby reducing the incidence of presenteeism and alleviating the psychological stress and trauma associated with their work.

## Data availability statement

The raw data supporting the conclusions of this article will be made available by the authors, without undue reservation.

## Ethics statement

Written informed consent was obtained from the individual(s) for the publication of any potentially identifiable images or data included in this article.

## Author contributions

DL: writing—original draft preparation. YL and TY: conceptualization and methodology. XK and SL: investigation. JY: supervision. AZ: writing—reviewing and editing. DL and YL: contributed in writing and revising the article. All authors contributed to the article and approved the submitted version.
